# Submaximal fitness and mortality risk reduction in coronary heart disease: a retrospective cohort study of community-based exercise rehabilitation

**DOI:** 10.1136/bmjopen-2016-011125

**Published:** 2016-06-29

**Authors:** Claire Taylor, Costas Tsakirides, James Moxon, James William Moxon, Michael Dudfield, Klaus K Witte, Lee Ingle, Sean Carroll

**Affiliations:** 1 Department of Sport, Health and Exercise Science, University of Hull, Hull, UK; 2 Carnegie School of Sport, Leeds Beckett University, Leeds, UK; 3 Burton Croft Surgery, Leeds, UK; 4 Retired General Practitioner, Leeds, UK; 5 Retired Fitness Development Officer, Sports Development, Leeds Leisure Services, Leeds, UK; 6 Division of Cardiovascular and Diabetes Research, University of Leeds, Leeds, UK

**Keywords:** cardiac rehabilitation, submaximal exercise testing, cardiorespiratory fitness, CHD, survival

## Abstract

**Objectives:**

To examine the association between submaximal cardiorespiratory fitness (sCRF) and all-cause mortality in a cardiac rehabilitation (CR) cohort.

**Design:**

Retrospective cohort study of participants entering CR between 26 May 1993 and 16 October 2006, followed up to 1 November 2013 (median 14 years, range 1.2–19.4 years).

**Setting:**

A community-based CR exercise programme in Leeds, West Yorkshire, UK.

**Participants:**

A cohort of 534 men (76%) and 136 women with a clinical diagnosis of coronary heart disease (CHD), aged 22–82 years, attending CR were evaluated for the association between baseline sCRF and all-cause mortality. 416 participants with an exercise test following CR (median 14 weeks) were examined for changes in sCRF and all-cause mortality.

**Main outcome measures:**

All-cause mortality and change in sCRF expressed in estimated metabolic equivalents (METs).

**Results:**

Baseline sCRF was a strong predictor of all-cause mortality; compared to the lowest sCRF group (<5 METs for women and <6 METs for men), mortality risk was 41% lower in those with moderate sCRF (HR 0.59; 95% CI 0.42 to 0.83) and 60% lower (HR 0.40; 95% CI 0.25 to 0.64) in those with higher sCRF levels (≥7 METs women and ≥8 METs for men). Although improvement in sCRF at 14 weeks was not associated with a significant mortality risk reduction (HR 0.91; 95% CI 0.79 to 1.06) for the whole cohort, in those with the lowest sCRF (and highest all-cause mortality) at baseline, each 1-MET improvement was associated with a 27% age-adjusted reduction in mortality risk (HR 0.73; 95% CI 0.57 to 0.94).

**Conclusions:**

Higher baseline sCRF is associated with a reduced risk of all-cause mortality over 14 years in adults with CHD. Improving fitness through exercise-based CR is associated with significant risk reduction for the least fit.

**Strengths and limitations of this study**

- Most detailed evaluation of prognostic risk associated with baseline and short-term submaximal cardiorespiratory fitness (sCRF) changes following exercise-based cardiac rehabilitation (CR) within a UK cohort.
- Longest complete follow-up of survival data among unselected male and female participants within a community-based CR cohort.
- Risk estimates for all-cause mortality associated with sCRF adjusted for numerous confounders, including coronary heart disease diagnosis, cardiovascular disease and other comorbidities, exercise test abnormalities, secondary prevention medications and self-report physical activity.
- Use of submaximal exercise test termination criteria and exercise capacity assessment is limited compared to direct measurement (or estimation) of maximal oxygen uptake (VO_2_ max) from maximal exercise testing.
- Reasons for non-completion of CR not routinely monitored or recorded.

## Introduction

The prognostic importance of cardiorespiratory fitness (CRF), as determined by direct measurement or prediction of maximal oxygen uptake (VO_2_ max), in men and women with coronary heart disease (CHD) has been widely reported within epidemiological studies.^1–4^ The fitness-mortality relationship has been shown to be non-linear, with the greatest mortality risk observed among the least fit.^5–7^ Findings from the Cardiac Wellness Institute of Calgary have recently demonstrated that each 1-metabolic equivalents (MET) improvement in CRF achieved during cardiac rehabilitation (CR) is associated with significant additional risk reduction for patients with the lowest CRF levels.^6^ Accordingly, improving low levels of CRF in patients undertaking exercise-based CR is potentially an important therapeutic goal.

Studies examining mortality and CRF outcomes from UK CR programmes are sparse.^8^ ^9^ The largest randomised clinical trial of CR in the UK reported no survival benefit from CR, though importantly, it did not consider changes in patients' CRF levels.^10^ A previous international meta-analysis has suggested mean CRF improvements of 1.6 METs following CR, though it did not include data from the UK.^11^ In contrast, the estimated mean MET change determined from submaximal testing during supervised exercise training across four UK centres was 0.5 MET;^8^ though the exercise prescription in these centres was conservative by European standards.^12^ Thus, whether this magnitude of CRF change is representative of typical UK CR is not known.

To the best of our knowledge, only one recent study has investigated the factors predicting long-term survival (including the role of CRF) within the UK CR setting.^9^ However, importantly, it did not quantify CRF change or associated mortality risk estimates across the CRF distribution. An improved understanding of these associations may help UK clinicians and CR practitioners to better determine the efficacy of CR for mortality risk reduction and the clinical benefit of CRF improvement for patients. The objectives of this study were therefore to:^1^ examine the association between baseline CRF and all-cause mortality from routine submaximal exercise test data collected from a community exercise-based CR cohort;^2^ evaluate short-term changes in submaximal CRF (sCRF) following CR; and^3^ examine the mortality risk reduction associated with sCRF changes, with specific reference to those with the lowest sCRF levels at CR entry.

## Methods

### Setting and participants

This is a retrospective cohort study of participants entering a community-based CR exercise programme, ‘Heart Watch’, delivered by local council leisure services in Leeds, UK, between 26 May 1993 and 16 October 2006. All participants were enrolled with a clinical diagnosis of CHD and classified as follows: previous myocardial infarction (MI), previous revascularisation therapy (including coronary artery bypass grafting (CABG) or percutaneous coronary intervention (PCI). Programme enrolment was made on the basis of general practitioner or consultant cardiologist/CR nurse referral. Participants were clinically stable and discharged from hospital for a minimum of 12 weeks.

### Data sources and measurement

Mortality status was ascertained from date of death from clinical databases (Leeds Teaching Hospitals' National Health Service (NHS) Trust) that use established tagging procedures provided by NHS registers in England. These databases were accessed using personal identifiers (full name, date of birth, postcode) to provide secure data linkage with the Heart Watch data registry.

The Heart Watch database has captured medical and exercise testing and training data for all participants enrolled in the programme since 1993. At enrolment, routinely collected data include: date of entry, age, sex, postcode, marital status, aetiology of CHD disease, family history, previous cardiac history and procedures (eg, CABG, PCI), cardioprotective medications, smoking status (categorised as never, <10 years and >10 years cessation and current), and comorbidities including: diabetes mellitus, hypertension, chronic heart failure, cerebrovascular disease, valvular disease and peripheral vascular disease. Cardiovascular risk factor measurements include waist circumference, body mass index (BMI), blood pressure and lipid profile. New paragraph required here Body mass and height were recorded and BMI was calculated as mass (kg) divided by stature squared (m^2^). Manual arm-cuff sphygmomanometry was used for all resting blood pressure assessments. sCRF measurements included: exercise duration, resting and exercise heart rates (HR), rating of perceived exertion (RPE) using the Borg 6–20 scale,^13^ symptoms (dyspnoea and/or angina) and electrocardiographic responses, recorded throughout submaximal exercise testing. An exercise test date was used to adjust for temporal trends in pharmacological therapy and treatment of CHD. Exercise adherence to CR and self-reported physical activity were ascertained from attendance registers and participant interview at the retest. Participants who underwent exercise testing at baseline but did not return for the retest (scheduled after 12 weeks of CR) were identified as ‘non-completers’. These participants typically attended no or few CR sessions.

sCRF was defined in METs (1-MET ≈3.5 mL/kg/min), estimated from the final treadmill speed and grade during submaximal exercise testing.^14^ The majority of exercise tests were conducted using a progressive incremental treadmill walking protocol.^15^ Participants exercised up to an 85% age-predicted maximal heart rate (or RPE 16–17), unless clinically contraindicated.^16^ For participants exercising on the cycle ergometer (12%), exercise time and final work rate (Watts) were recorded and the latter converted to estimated METs.^17^ These protocols were adopted as directly determined VO_2_ and HR values for corresponding stages on the treadmill and cycle ergometer have been shown to be highly correlated (r=0.94 and r=0.89 for VO_2_ and HR, respectively), indicating comparability between test modalities.^15^ Handrail support during treadmill testing was discouraged. Participants' medications were not changed before exercise testing and the same test modality used pre-CR and post-CR.

There is no widely accepted clinical categorisation of the sCRF phenotype in adults with CHD. In previous epidemiological analyses, low, moderate and high CRF has been defined as the lower 20%, next 40% and upper 40% of the exercise duration distribution.^18^ ^19^ Sex-specific distributions of exercise test time in the present study were assessed and the following categories for low, moderate and higher sCRF determined; men <8 min, 8–10 min and ≥11 min; women <5 min, 5–8 min and ≥9 min. In equivalent MET values, cut-offs were <6 METS and ≥8 METs (men), and <5 METs and ≥7 METS (women). For comparison, sCRF was also analysed using cut-offs published previously.^2^ ^7^

### Description of the CR programme

The exercise training component of the Heart Watch CR programme has been fully described elsewhere.^20^ All exercise sessions were formally supervised by exercise instructors in a structured setting up to 5 days per week. Participants received a mixed (aerobic and resistance) circuit-based exercise of 24 min duration, with a 15 min warm-up and 15 min cool-down. Aerobic exercises included floor and treadmill walking, stepping, leg cycling, arm-leg cycling and rowing ergometry. Resistance and floor-based exercise sets involved eight exercises performed for 30 s each. Participants were strongly encouraged to attend classes on three non-consecutive days and walk 30 min per day outside of the programme. On the basis of resting and final HR and RPE from the exercise test, participants were prescribed individual target heart rate training (between 40 and 85% heart rate reserve) with Polar heart rate monitors. They were closely supervised by exercise instructors, ensuring good adherence to exercise prescription.

### Statistical analysis

Measures of central tendency and dispersion are reported as mean and SD unless specified. All participant characteristics at entry to CR (table 1) were compared across sCRF categories using the χ^2^ test for categorical variables and one-way analysis of variance (ANOVA) for continuous variables (Kruskal-Wallis non-parametric equivalent where appropriate). Characteristics of programme completers and non-completers were compared using independent t-tests. A mixed-model two-way ANOVA was used to assess differences in exercise variables from baseline to follow-up (within-subject factors) and between sCRF categories (between-subject factors) with appropriate post hoc procedures. For variables that were log transformed before modelling, the mean presented is the back transformed mean.

**Table 1:** Baseline demographic and clinical characteristics in n=670 participants stratified by sCRF category

Characteristic	Low sCRF (n=128)†	Moderate sCRF (n=317)	Higher sCRF (n=225)‡	p Value
Age (years)	64 (9.7)	61 (8.7)	55 (8.6)	<0.0005
Sex, male (%)	79	77	84	0.196
Elderly (age >75 years) (%)	12	3	0.0	<0.0005
Married/living with partner (%)	76	82	83	0.221
Previous MI (%)	31	32	42	0.029
Previous CABG (%)	43	46	35	0.052
Previous PCI (%)	13	14	16	0.575
Previous Angina (%)	27	29	20	0.050
Diabetes mellitus (%)	22	14	5	<0.0005
Hypertension (%)	11	16	14	0.461
Premature family history (%)	39	37	38	0.886
Chronic heart failure (%)	3	2	0.4	0.140
COPD (%)	0.8	2	1	0.804
Peripheral vascular disease (%)	3	0.9	0.4	0.071
Valvular disease (%)	10	5	4	0.053
Cerebrovascular disease (%)	0.0	1	0.4	0.305
BMI >30 (kg/m^2^) (%)	35	32	20	0.002
Waist circumference (cm)	100 (14)	98 (12)	95 (10)	<0.0005
HDL cholesterol (mmol/L)	1.2 (0.3)	1.2 (0.3)	1.2 (0.3)	0.508
Total cholesterol (mmol/L)	5.3 (1.1)	5.6 (1.2)	5.5 (1.1)	0.092
TC/HDL ratio	4.8 (1.7)	4.9 (1.5)	4.8 (1.5)	0.828
LDL cholesterol (mmol/L)	3.3 (1.1)	3.5 (1.1)	3.5 (1.0)	0.198
Triglycerides (mmol/L) (LOG10)	1.5	1.6	1.5	0.212
Previous (<10 years/current smoker) (%)	36	35	40	0.467
Physically inactive (%)	41	29	20	<0.0005
SBP rest (mm Hg)	144 (21)	146 (22)	138 (20)	<0.0005
DBP rest (mm Hg)	83 (11)	86 (12)	86 (11)	0.399
HR Rest (bpm)	75 (16)	72 (16)	63 (13)	0.007
ACE Inhibitor (%)	24	22	16	0.170
Antiplatelet therapy (%)	68	75	77	0.138
β-Blocker (%)	34	39	52	0.002
Diuretic (%)	40	25	9	<0.0005
Statin (%)	41	37	39	0.630
Exercise test characteristics				
Test time (min), median (IQR)	6 (4–6)	8 (8–10)	12 (12–14)	<0.0005
Power output (Watts)	71 (16)	106 (17)	145 (10)	<0.0005
Exercise mode (treadmill) (%)	87	89	89	0.794
Peak HR (% APMHR)	94 (14)	95 (9)	92 (8)	0.008
Peak HR β-blocked (% APMHR)	92 (21)	93 (15)	95 (12)	0.219
Peak RPE	14 (1.8)	15 (1.7)	15 (1.7)	<0.0005
Peak RPE (β-blocked)	14 (1.7)	16 (1.4)	15 (1.5)	<0.0005
Estimated METs, median (IQR)	5 (3.7–5)	6.1 (6.1–7)	8.3 (8.3–9.6)	<0.0005
Positive exercise test (ECG) (%)	21	13	5	<0.0005

Results presented as mean (SD) unless otherwise stated.

†Entry sCRF level <6 METs (men) and <5 METs (women).

‡Entry sCRF level ≥8 METs and ≥7 METs (women).

APMHR, age-predicted maximum heart rate; BMI, body mass index; bpm, beats per minute; CABG, coronary artery bypass graft; COPD, chronic obstructive pulmonary disease; DBP, diastolic blood pressure; ECG, electrocardiogram; HDL, high-density lipoprotein; HR, heart rate; LDL, low-density lipoprotein; METs, metabolic equivalents; MI, myocardial infarction; PCI, percutaneous coronary intervention; SBP, systolic blood pressure; TC/HDL, total cholesterol high-density lipoprotein cholesterol ratio.

The primary outcome measure was all-cause mortality at 14 years. Cox proportional hazard models were used to determine the association between sCRF and survival.^21^ Clinically relevant variables with a p value<0.05 from univariate analysis were included in the multivariate model. Logistic regression (backward stepwise) was performed and HRs with 95% confidence intervals (95% CI) were reported. Age at initial test was adjusted in years as a continuous variable. Estimated METs was entered as a continuous variable and a categorical variable. Receiver-operating characteristic (ROC) curves were constructed to determine an optimal MET level threshold for all-cause mortality.

A parsimonious approach to variable selection was taken for multivariate analyses with 10 events per independent variable analysed for model fit.^22^ ^23^ No significant collinearity (assessed using variance inflation factors) was observed between variables selected for models (all <1.5). Analyses were adjusted for age, waist circumference and total cholesterol high-density lipoprotein cholesterol (TC/HDL-c) ratio (continuous variables); cardioprotective medications (antiplatelet therapy, ACE inhibitors, (β)-blockers, diuretics and statins), physical inactivity, diabetes, other CVD (chronic heart failure, valvular disease, arrhythmia, vascular disease), marital status, exercise ECG test result, date of entry to CR (median split) and exercise test mode (all categorical variables).

The χ^2^ statistic was used to assess the statistical importance of each parameter for prediction of all-cause mortality risk. A Kaplan-Meier survival plot was constructed, with a log rank test to compare the time-dependent occurrence of death in the groups stratified according to sCRF categories at CR entry. The proportional hazards assumption was verified as proportional over time with a log(-log(survival)) plot versus log(time) plot for all variables. Continuous variables were categorised into groups and model goodness-of-fit shown to be well calibrated by the Hosmer-Lemeshow test (p>0.05).

For variables of primary interest, no data were missing from baseline and follow-up assessments. Missing data in other variables accounted for <5% of the final sample. Analyses were performed using SPSS V.23.0 software (SPSS Inc, Chicago, Illinois, USA).

## Results

Nine hundred and ninety-four participants (mean age 59.5; 76% male) were referred to CR. Participants who died within 12 months of their baseline assessment (n=7), those without complete follow-up (n=315) and those without a valid baseline exercise test (n=2) were excluded, leaving a final study population of n=670. All participants underwent baseline submaximal exercise testing and medical assessment and had a minimum of 1 year of follow-up. Follow-up was complete to 1 November 2013.

Analysis of baseline sCRF and survival was based on 670 participants. Of these, n=465 (69%) also attended a follow-up assessment after 14 weeks (median) CR. The analysis of sCRF change is therefore based on 465 participants with complete data from 2 exercise tests. Participants without a follow-up test (‘non-completers’; n=205, 31%) were subsequently compared to completers in subgroup analysis. Female participants (n=136, 20%) were excluded from ROC curve analysis as the sample size did not support statistical analysis.

### Participant characteristics

Table 1 describes the demographic and clinical characteristics of the study participants based on sCRF at entry to CR. One hundred and twenty-eight (19%) participants were classified as having a low sCRF level, 317 (47%) a moderate sCRF level and 225 (34%) a higher sCRF level. Participants with low sCRF were older and had a higher prevalence of positive exercise tests (ECG abnormality/angina/severe dyspnoea), comorbidities (including diabetes and obesity) and self-reported physical inactivity. Resting HR was also higher. There was a higher prevalence of diuretic therapy use and lower use of β-blocker therapy. Similar peak HR values on exercise testing were attained for those on β-blocker therapy but were higher for low and moderate sCRF groups in those not on β-blockers. Peak ratings of RPE in the low sCRF group tended to be lower than in participants with moderate or higher sCRF. Secondary prevention medications prescribed in the cohort overall were: β-blockers 48%, antiplatelet therapy (mainly aspirin) 81%, statin therapy 38%, diuretics 23% and ACE inhibitors 20%. These were consistent for all participants between CR entry and follow-up.

Two-hundred and five participants (31%) in the study cohort did not attend initial reassessment following CR. Reasons for non-completion were not recorded; however, participants not completing were younger (58±10 vs 60±9 years) and less likely to have undergone previous CABG surgery. ‘Non-completers’ were also more likely to be physically inactive (36% vs 24%) and a current/previous smoker (all p<0.05). Since groups were similar with respect to survival and all other baseline characteristics, including sCRF, both groups were included in survival analysis.

### sCRF at CR entry and all-cause mortality risk

Clinical predictors of all-cause mortality from Cox proportional hazards models are presented in table 2. After a median follow-up of 14 years (range, 1.2 years–19.4 years), 206 deaths (31%) were recorded: 54% in the low sCRF group (n=69 deaths), 31% in the moderate sCRF group (99=deaths) and 17% in the higher sCRF group (n=38 deaths). Baseline sCRF was 7.1**±**1.8 METs among survivors and 6.1±1.7 METs in decedents (p<0.0005). Significant univariate predictors of all-cause death were low baseline sCRF level, age, abnormal exercise test response, co-existing CVD disorders, physical inactivity and taking specific cardioprotective medication (diuretics, statin and ACE-inhibitors).

**Table 2:** Survival estimates from multivariate Cox regression analysis for all-cause mortality

Variable	HR (95% CI)	χ^2^	p Value
All-cause mortality (n=670)			
Other CVD	2.07 (1.45 to 2.93)	16.4	<0.0005
Age	1.03 (1.02 to 1.05)	15.1	<0.0005
Higher sCRF†	0.40 (0.25 to 0.64)	14.7	<0.0005
ACE-inhibitor use	1.85 (1.34 to 2.57)	13.8	<0.0005
Date of CR entry	1.74 (1.25 to 2.41)	10.9	0.001
Statin use	1.62 (1.18 to 2.22)	8.9	0.003
Antiplatelet therapy use	0.66 (0.48 to 0.92)	5.9	0.015
Married/living with partner	0.66 (0.47 to 0.92)	5.9	0.016
Diuretic use	1.35 (0.97 to 1.87)	3.2	0.075

Univariate predictors: diabetes, physical inactivity, exercise test modality, entry METs, TC/HDL-c ratio, waist circumference, resting heart rate were no longer significant predictors in the multivariate model.

†Entry sCRF level ≥8 METs (men) and ≥7 METs (women).

CVD, cardiovascular disease; CR, cardiac rehabilitation; METs, metabolic equivalents; TC/HDL-c total cholesterol high-density lipoprotein-cholesterol ratio; sCRF, submaximal cardiorespiratory fitness.

Sex, smoking status (including never, former or current), previous MI, body mass index, β-blocker medication use, resting systolic blood pressure, non-completer status and indices of multiple social deprivation were not univariate predictors of all-cause mortality (p>0.05) in this cohort.

**Figure 1:**
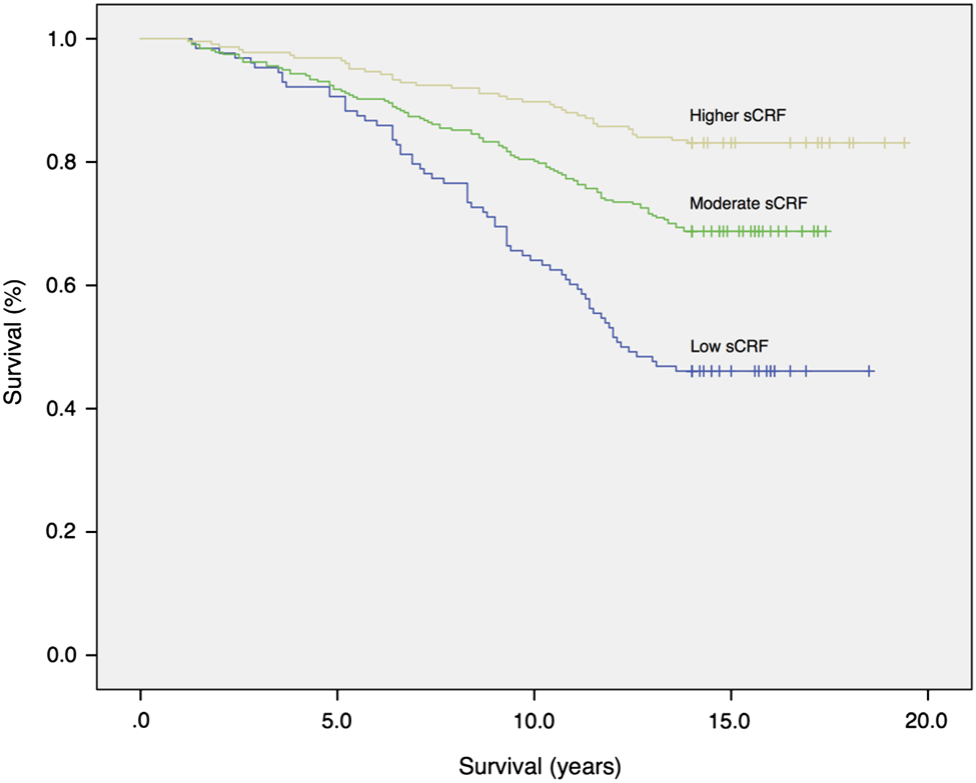
Kaplan-Meier plot showing risk of all-cause mortality in participants stratified by baseline sCRF (log-rank test; p<0.0005). sCRF, submaximal cardiorespiratory fitness.

For the cohort overall, age-adjusted mortality risk was 17% lower for each 1-MET increment in sCRF at CR entry (HR 0.83; 95% CI 0.76 to 0.90). In multivariate-adjusted analysis, mortality risk was 11% lower per 1-MET increment (HR 0.89; 95% CI 0.81 to 0.98). Relative mortality risks across sCRF categories are presented in table 3 and survival plot in figure 1. Compared to the low sCRF group, all models (unadjusted, age-adjusted and multivariate-adjusted) demonstrated significantly lower HRs for moderate and higher sCRF groups. Mortality risk was 41% lower for those with moderate sCRF (HR 0.59; 95% CI 0.42 to 0.83), and 60% lower for those with higher sCRF (HR 0.40; 95% CI 0.25 to 0.64), representing a more than two-fold increase in relative risk of death for participants with low sCRF compared to their higher fit counterparts.

**Table 3:** Comparison of HRs for all-cause mortality between low, moderate and higher sCRF participants at CR entry (n=670)

All-cause mortality (n=206 deaths)	Low sCRF* (referent) (n=128)	Moderate sCRF HR (95% CI) (n=317)	p Value	Higher sCRF† HR (95% CI) (n=225)	p Value
Model 1: unadjusted	1.00	0.50 (0.37 to 0.68)	<0.0005	0.25 (0.17 to 0.37)	<0.0005
Model 2: adjusted for age	1.00	0.55 (0.40 to 0.75)	<0.0005	0.35 (0.23 to 0.53)	<0.0005
Model 3: adjusted for covariates	1.00	0.59 (0.42 to 0.83)	0.002	0.40 (0.25 to 0.64)	<0.0005

Model 3 covariates were as follows: age; ACE-inhibitor, statin, diuretic, antiplatelet therapy use; diabetes, other CVD, waist circumference, TC/HDL ratio, date of CR entry, marital status, physical inactivity, resting heart rate, exercise test mode and negative exercise test (ECG).

*Entry sCRF level <6 METs (men) and <5 METs (women) at CR entry.

†Entry sCRF level ≥8 METs (men) and ≥7 METs (women) at CR entry.

CVD, cardio vascular disease; CR, cardiac rehabilitation; METs, metabolic equivalents; TC/HDL, total cholesterol high-density lipoprotein ratio; sCRF, submaximal cardiorespiratory fitness.

In secondary analysis, thresholds used previously to examine the prognostic role of CRF were compared to our distributional analysis (see online supplementary table S1). Univariate and multivariate analysis confirmed sCRF as an independent predictor of all-cause mortality.

10.1136/bmjopen-2016-011125.supp1 Supplementary Table 1 Comparison of hazard ratios (HR) for all-cause mortality between low, moderate and higher sCRF groups at CR entry (n=670)

### sCRF change and all-cause mortality risk

Of the 465 participants who attended baseline and follow-up assessment, a further n=49 were excluded from the subgroup analysis of sCRF change following CR. Reassessments for these participants were delayed, and thus exercise test data were potentially unrepresentative of sCRF status following CR. Data presented are from n=416 participants with two valid exercise tests pre-CR and post-CR. The mean weekly frequency of exercise training (2±1 sessions) and the total number of sessions attended (28±9 sessions were not significantly different between low, moderate, and higher sCRF groups (both p>0.05).

Following 14 weeks of exercise-based CR, sCRF values improved across the cohort (0.82 MET improvement; p<0.0005), with no significant difference between males and females (0.83 vs 0.79 METs, respectively). The majority of participants improved or at least maintained their sCRF classification at follow-up (table 4). Notably, 69% with low baseline fitness and 45% with moderate baseline fitness improved classification.

**Table 4:** sCRF reclassification from baseline to follow-up after 14 weeks CR (n=416)

	sCRF classification at follow-up after CR (%)		
sCRF classification at CR entry	Improved* sCRF	Maintained† sCRF	Worsened sCRF
Low sCRF (n=74)	69	31	−
Moderate sCRF (n=196)	45	53	2
Higher sCRF (n=146)	−	91	9

*Defined as improving sCRF to a higher sCRF category between the baseline and follow-up test.

†Defined as no change in the sCRF category from the baseline to the follow-up test.

CR, cardiac rehabilitation; sCRF, submaximal cardiorespiratory fitness.

However, there was a differential mean sCRF improvement (based on estimated METs and exercise time) across sCRF groups (table 5). A significantly higher mean improvement was evident in the low sCRF category (1.36 METs; 95% CI 1.07 to 1.64) compared to both the moderate (0.99 METs; 95% CI 0.85 to 1.13) and higher sCRF groups (0.31 METs; 95% CI 0.12 to 0.50). There was no difference in peak HR across groups. Among participants in the low sCRF category, each 1-MET improvement was associated with a 27% lower age-adjusted risk of mortality (HR 0.73; 95% CI 0.57 to 0.94). In contrast, there was no significant risk reduction associated with estimated MET gain for moderately fit (HR 0.77; 95% CI 0.59 to 1.01) or higher-fit (HR 0.95; 95% CI 0.68 to 1.33) groups. The highest relative risk of mortality was observed in participants classified as low fit at CR entry and follow-up (n=23; HR 7.94; 95% CI 4.28 to 14.75).

**Table 5:** Mean changes in exercise test variables from baseline to follow-up after 14 weeks CR (n=416)

Exercise test characteristics	Low sCRF† (n=76)	Moderate sCRF (n=196)	Higher sCRF‡ (n=146)	p Value
Change in METs	1.36 (1.07 to 1.64)	0.99 (0.85 to 1.13)	0.31 (0.12 to 0.50)	<0.0005
Change in exercise time (min)	2.4 (1.9 to 3.0)	1.6 (1.4 to 1.8)	0.5 (0.2 to 0.8)	<0.0005
Change in power output (W)	38 (26 to 53)	20 (11 to 26)	10 (0.7 to 20)	0.122
Change in peak HR (bpm)	−1 (−1 to 0)	1 (1 to 1)	0 (0 to 0)	0.761
Change in peak HR (β-blocked)	5 (5 to 6)	4 (4 to 4)	4 (3 to 4)	0.786

†Entry sCRF level <6 METs (men) and <5 METs (women) at CR entry.

‡Entry sCRF level ≥8 METs (men) and ≥7 METs (women) at CR entry.

bpm, beats per minute; CR, cardiac rehabilitation; sCRF, submaximal cardiorespiratory fitness; METs, metabolic equivalents; W, Watts.

To determine the optimal discriminatory accuracy of sCRF for predicting all-cause mortality in this cohort, a ROC curve analysis was also performed (restricted to males; n=534). The optimal sCRF threshold was 6.5 METs (area under the curve 0.71 (0.66 to 0.76); sensitivity 67% and specificity 63%). This threshold was used to define lower (≤6.5 METs) and higher (>6.5 METs) sCRF categories. After age, a higher sCRF level was the highest ranking predictor of all-cause mortality (table 6). Self-reported physical inactivity at CR entry was a multivariate predictor in male-only analyses.

**Table 6:** Survival estimates from multivariate Cox regression analysis for all-cause mortality among males (n=534)

Variable	HR (95% CI)	χ^2^	p Value
All-cause mortality			
Age	1.04 (1.02 to 1.06)	16.5	<0.0005
Higher sCRF†	0.39 (0.23 to 0.66)	14.7	<0.0005
Other CVD	1.99 (1.37 to 2.90)	12.8	<0.0005
Statin use	1.78 (1.29 to 2.47)	12.2	<0.0005
Antiplatelet therapy use	0.58 (0.41 to 0.82)	9.6	0.002
Diuretic therapy use	1.71 (1.20 to 2.45)	8.7	0.003
ACE-inhibitor use	1.53 (1.07 to 2.20)	5.5	0.020
Physically inactive‡	1.46 (1.04 to 2.05)	4.7	0.030

†Entry sCRF level >6.5 METs.

‡Self-reported sedentary.

CVD, cardiovascular disease; sCRF, submaximal cardiorespiratory fitness; METs, metabolic equivalents.

## Discussion

To the best of our knowledge, this is the most detailed evaluation of prognostic risk associated with sCRF at entry to CR and the first UK study to examine the relationship between short-term sCRF changes and all-cause mortality among participants attending a community-based programme. Our data demonstrate that initial sCRF level was the strongest modifiable predictor of long-term survival, surpassed only by older age and co-existing CVD. This relationship remained following adjustment for a number of important confounders not previously considered in UK CR studies, including coronary risk factors and self-reported physical inactivity. Moreover, for the lowest-fit individuals, we report a quantifiable reduction in all-cause mortality risk per MET increase in sCRF achieved during exercise-based CR. This may be clinically important given the higher baseline mortality risk of this group compared to their higher fit counterparts.

Our data compare favourably with previous international studies employing maximal exercise testing and demonstrating an association between CRF and prognosis in patients with CHD.^1^ ^2^ ^4^ In a study of 2812 patients entering CR between 1996 and 2004 by Keteyian *et al*,^4^ each 1 mL/kg/min increment in the estimated VO_2_ peak at baseline demonstrated a ∼15% lower risk for all-cause mortality. When extrapolated to 1-MET (HR 0.85^3.5^=0.57), this represents a reduction in risk equivalent to 43% per MET. In a more recent study of patients with CVD referred for clinical exercise testing, Mandic *et al*^5^ estimated a more modest 11% reduction in all-cause mortality risk with each 1-MET advantage in VO_2_ peak. The variance in risk estimates attributed to CRF between observational investigations is most likely due to differences in cohort characteristics, medical and secondary prevention treatments and exercise testing modalities (ie, maximal vs submaximal exercise protocols). It is also likely to reflect the variability in thresholds used to define low and high CRF categories,^1^ ^5^ ^7^ given that there is currently no consensus for the clinical categorisation of CRF in patients with CHD.

Our data extend findings from the only other published study to examine the fitness-mortality relationship in patients undertaking CR within the UK.^9^ In that study, CRF was estimated from maximal testing in a cohort of predominantly post-MI patients (86% men, age 61 years) over a shorter follow-up (mean 10.7 years). Low CRF at entry and CRF improvement during once-or-twice weekly CR were strong predictors of survival. However, since neither relationship was expressed in the context of survival benefit per MET increment in exercise capacity, it is not possible to quantify the change in fitness comparable with international studies. The overall improvement in sCRF within our cohort (0.82 MET) is analogous to that estimated during maximal testing by Barons *et al* (1.08 METs) and congruent with the ∼0.5 MET improvement estimated by Sandercock *et al*^8^ in 950 patients undergoing submaximal testing across four UK CR centres.

To the best of our knowledge, this is the first UK study to report a quantifiable dose–response to CR exercise training, with a 27% reduction in all-cause mortality risk per MET increase achieved by the lowest fit. It substantiates the findings of Martin *et al*^6^ from the Cardiac Wellness Institute of Calgary who estimated 1.41 METs, 1.01 METs and 0.80 MET improvements from maximal testing, for low, moderate and high CRF groups, respectively in their large cohort (76% men, age 60 years). These investigators also found that each MET gain during CR was associated with a 30% point reduction in mortality risk for the least fit patients (<5 METs). This is similar to the 27% age-adjusted risk reduction in the lowest sex-specific sCRF groups (<5 METs women, <6 METs men) we report. In contrast, Barons *et al*^9^ reported no significant risk reduction associated with improvement from low to moderate fitness during CR. Others assessing the relationship between submaximal exercise training workload in CR and medium-term survival (mean 4.4 years) have estimated a 28% age-adjusted reduction in mortality risk per MET increase over 12 weeks (36 sessions).^24^

It is noted that medical therapies used in several previous observational studies either antedated the contemporary use of cardioprotective pharmacotherapies, or patients were not treated rigorously with available secondary preventative medications. Our finding that certain cardioprotective medications, notably ACE inhibitor;^1^ ^9^ diuretics^1^ ^3^ and statins,^5^ were independent adverse predictors of all-cause mortality has been shown previously. Higher CRF associated with improved survival in hyperlipidaemic men and women has been shown to affect the positive risk reduction from statin therapy in those with established CHD within the Henry Ford Exercise Testing cohort.^25^ There are limited previous data to support our observation that β-blockade does not interfere with the prognostic significance of low exercise capacity in patients with CR.^7^ The observational findings of adverse risks associated with cardioprotective therapies are contrary to strong randomised controlled trial evidence^26–28^ demonstrating the efficacy of these treatments for secondary prevention of CHD and may reflect differences in severity of underlying disease and prevalence of comorbidities among individuals receiving these treatments.

This is a retrospective study with a number of limitations. The use of prespecified (submaximal) test termination criteria (attainment of 85% age-predicted maximal heart rate) is acknowledged to be inferior to the measurement (or estimation) of VO_2_ peak and equivalent MET levels from maximal testing, and so our results do not reflect participants' individualised peak aerobic capacity. Further, while the MET is a widely used physiological metric, its limitations are acknowledged.^29^ Unmeasured confounders remain a limitation associated with all observational study designs and measures that may have been relevant to the current analysis, such as compliance with home exercise during CR, were not captured in the Heart Watch database. In addition, we are not able to report why CR non-completers left the programme, as precise reasons were not routinely recorded.

## Conclusions

Our results demonstrate the strong prognostic value of sCRF and the risk reduction associated with higher levels of sCRF in men and women starting exercise-based CR, irrespective of other important markers of risk. Importantly, for the lowest-fit individuals, increasing sCRF through exercise-based CR may be associated with significant additional survival benefit, reinforcing the clinical importance of moving patients out of this higher risk group. These patients may also benefit most from early identification and closer monitoring by CR staff through serial exercise testing.

Accessible data from submaximal exercise test performance (with electrocardiographic analysis) may yield important prognostic information for the assessment of sCRF and the risk stratification of patients undertaking community-based CR within the UK. These data may help clinicians and CR practitioners improve the design of structured, supervised exercise training services tailored to maximise CRF gains for the least fit patients and thus, improve long-term survival following CR.
