# Supplementary material for: Submaximal fitness and mortality risk reduction in coronary heart disease: a retrospective cohort study of community-based exercise rehabilitation
Source: BMJ Open. 2016 Jun 29;6(6):e011125. doi: 10.1136/bmjopen-2016-011125 (PMC4932274; doi:10.1136/bmjopen-2016-011125)
Supplement: Supplementary Table 1 — Comparison of hazard ratios (HR) for all-cause mortality between low, moderate and higher sCRF groups at CR entry (n=670) [file bmjopen-2016-011125supp_table.pdf]

Supplementary Table 1. Comparison of hazard ratios (HR) for all-cause mortality between low, moderate and higher sCRF groups at CR entry (n=670)

**All-cause mortality (n=206 deaths)**	**High sCRF † (referent) (n=202)**	**Moderate sCRF HR (95% CI) (n=404)**	***p* Value**	**Low sCRF ‡ HR (95% CI) (n=64)**	***p* Value**
Model 1: unadjusted	1.00	2.61 (1.77 to 3.85)	<.0005*	4.16 (2.55 to 6.80)	<.0005*
Model 2: adjusted for age	1.00	2.01 (1.34 to 3.00)	.001*	2.63 (1.55 to 4.45)	<.0005*
Model 3: adjusted for covariates	1.00	1.81 (1.16 to 2.81)	.008*	1.92 (1.07 to 3.45)	<.029*

† entry sCRF level > 8 METs (men) and > 6 MET (women) at CR entry ‡ entry sCRF level < 5 METs (men) and < 4 METs (women) at CR entry.
* significant at P<.05. Model 3 covariates were as follows: age; ACE-inhibitor, statin, diuretic, antiplatelet therapy use; diabetes, other CVD, waist circumference, TC/HDL ratio, date of CR entry, marital status, physical inactivity, resting heart rate, exercise test mode and negative exercise test (ECG).
